# The Hospital Nursing Supplement

**Published:** 1895-12-07

**Authors:** 


### "The Hospital" Nursing Mirror.

Being the Extra Nursing Supplement of "The Hospital" Newspaper.

[Contributions for this Supplement should be addressed to the Editor, The Hospital, 428, Strand, London, W.C., and should have the word "Nursing" plainly written in left-hand top corner of the envelope.]

### News from the Nursing World.

### OUR NEEDLEWORK COMPETITION.

We hope that our readers are keeping well in mind the dates on which we are expecting their contributions to our annual needlework competition. We look forward with pleasure to receiving a large supply of warm things for the patients who spend Christmas Day in hospital wards. The prizes offered are: One guinea for the best flannel dressing gown, 10s. for best bed jacket (for man or woman), 10s. for best flannel shirt, 5s. for best flannel petticoat, 5s. for best over-petticoat, 5s. for best knitted socks, 2s. 6d. for second best pair, 2s. 6d. for best warm vest (for man or woman). Each parcel should be addressed "Needlework Competition," care of the Editor of The Hospital, 428, Strand, London, and should contain the name and permanent address of the sender, and reach the office on December 16th or 17th.

### NURSES FOR COLONISTS.

The provision of trained private nurses for those colonies where they are not yet established is the aim of a new scheme now under contemplation. Suggestions on the subject are invited by Mrs. Piggott, 29, Cadogan Terrace, S.W., who hopes that some practical plan to deal with the matter may be organised early in the coming year. At present it appears to be thought that two or more nurses should be engaged (at an annual salary) to go out to each colony desiring their services. Free passage and lodging are also to be provided for them, and the engagement is to be binding for three years. It is proposed to raise a fund for this purpose, and, no doubt, colonists will be expected to co-operate and to share the expenses. Probably those hospitals in the colonies which already train their own probationers will shortly be able to supply settlers with private nurses. These would be far better suited to work abroad than those accustomed to very different surroundings in England. Probably some of our colonial correspondents will be able to tell us in how many of our settlements they find trained nurses absolutely unattainable at present?

### WELL-MERITED GRATITUDE.

It is with pleasure that we record the cordial vote of thanks to the Workhouse Infirmary Nursing Association passed by the St. George's-in-the-East Board of Guardians. Special attention was called at a recent meeting to the progress in nursing which had been made at the infirmary by means of the association. The improvements being now well-established, it was resolved that the St. George's nursing staff no longer needs the assistance of the association, which has always so many pressing claims on its resources. The Guardians have therefore empowered the medical superintendent and matron to select suitable trained nurses, and also a certain number of probationers who will undergo a course of three years' training.

### PERSHORE COTTAGE HOSPITAL.

The pretty new cottage hospital at Pershore has at present accommodation for four patients, and the sanitary and domestic arrangements made for them seem altogether excellent. The proposed nursing staff appears, according to the local press, to include a matron, nurse, and probationer, which, at first sight, seems altogether disproportioned to the small number of patients. The matron is presumably a fully-trained nurse, and therefore, with the aid of a probationer, could certainly manage the little institution perfectly. Probably this is what she will really do; for the nurse, although nominally on the Cottage Hospital staff, is to do district nursing in the town. It may be quite feasible to arrange for a local district nurse to be lodged at a hospital as long as there is room to spare, but it is unwise to recken her expenses as part of those of the hospital. Her duties are quite distinct from it, and therefore her salary and maintenance should form a separate item in the annual report. The matron and probationer would, of course, form the actual staff of the hospital. It is possible that the press may have given the public an erroneous impression, which the committee will do well to correet. By starting free from debt, and by securing much local interest for the hospital, the committee have proved themselves able organisers, and no doubt the nursing department will eventually be as well administered as everything else in the new institution at Pershore, to which we cordially wish a long and prosperous career.

### CREWE MEMORIAL HOSPITAL.

The Crewe Memorial Cottage Hospital has been used by a goodly number of patients since its opening last summer, and it has already proved useful in many serious cases of illness. The nursing staff consists of a fully-trained matron with an assistant nurse, but as the latter is needed for night duty, the committee have decided also to employ a probationer. It is a pretty little hospital, with sixteen beds.

### DUTY OR DRUDGERY?

A Scotchman has favoured the *Christian World* with a letter concerning the work of hospital nurses which is of an amusing character, although apparently it is written in sober earnest. He owns that he disapproves of girls not being allowed "to dirty their delicate fingers with honest work," and boasts that every one of his own daughters knows how to clean grates and scrub floors as well as any of his servants. Thus far the Scotchman's ideas seem pre-eminently practical, but with regard to nurses' duties he appears less well informed. He seems to imagine that if the profession of nursing is adopted by women on account of the "good they might be to their fellow creatures," they should be absolved from what he is pleased to call "drudgery," such as he says he would be ashamed to ask his scullerymaid to undertake. This wholesale condemnation, however, is due to a misconception. The work of nurses in English training schools doubtless does not include cleaning of grates nor scrubbing of floors, but it is not found that good nurses apply the term "drudgery" to other services rendered by them to the sick.

### WALLSEND NURSING ASSOCIATION.

The first year's work and the first year's income of this association are alike satisfactory, for there is a balance in hand and a goodly record of cases visited. The district has been divided into sections, and the collector in charge of each has obtained a large number of small subscriptions. The Loan and Food Association appears to have been equally well managed, and a little money is still in hand. The services of Nurse Rudd have been gratefully received by 110 patients, to whom she has paid 1,778 visits in the last eleven months. The career of the Wallsend Nursing Association deserves to be a prosperous one.

### A NEW ASSOCIATION.

The District Nursing Association recently established at Pitlochry is designed to benefit all the sick poor residing within a radius of two miles of the town. The fully-trained nurse is to enter on her duties at once, and will doubtless prove of great value to the whole district during the present winter.

### ST. LAWRENCE'S CATHOLIC HOME.

It is pleasant to note the cordial reception accorded to the district nurses trained at St. Lawrence's Home, Dublin, who are now at work at various remote country places as well as in Dublin itself. More subscriptions are sorely needed, as the funds of the home are not by any means in due proportion to the expenditure. The committee congratulate themselves on the admirable zeal and devotion to duty which their superintendent, Miss St. Clair, has consistently exhibited. So much good work has been done already by the St. Lawrence's Home branch of the Queen's Jubilee Institute that it is to be hoped it will eventually secure the substantial support which it merits.

### A NURSES' HOME AT DUNDEE.

The present inadequate provision for the nursing staff at Dundee Infirmary is likely to be superseded by a separate home in which each nurse will have her bed-room, instead of sharing one with several others. A gift of £3,500 from the president of the infirmary (Mr. Dalgleish) has been volunteered for this excellent object

### QUEEN'S NURSES AT INVERNESS.

The Inverness branch of the Queen Victoria's Jubilee Institute has been established for four years, and it is hoped that its financial condition will permit of a second nurse being engaged in the course of the next twelve months. There is an increasing demand for the services of the present nurse, and her labours receive much kindly local encouragement. At the annual meeting, which was largely attended, the various speakers showed great enthusiasm towards the work of the Institute. Invalid comforts have been provided by ladies in the neighbourhood, and other acceptable aid has been rendered.

### PAUPERS AS PROBATIONERS.

It was proposed the other day by a Kentish Guardian, according to the local press, that inmates of the Minster Workhouse should be trained as nurses, and for this purpose "sensible, careful, and prudent" persons should be chosen. That these and other qualifications are desirable in all probationers everyone must agree, but few workhouse inmates are likely to possess them. Sensible, careful, and prudent people seldom become chargeable to the rates unless they are permanently incapacitated by disease or infirmity. At the meeting where this novel suggestion for training was mooted, it was decided by a majority of the board that one night nurse should be engaged to undertake the duties, which two doctors and the Local Government Board had already agreed could only be properly carried out by two!

### MORE REGISTRATION FOR NURSES.

Although the Royal British Nurses' Association has not succeeded in convincing the public, the medical profession, and those concerned in the training of nurses that registration, as conducted by them, has fulfilled the object of raising the status of nurses and protecting the public efficiently, there are still many persons who think that some registration, such as State registration, is desirable. The Council of the British Medical Association is now taking the matter up, and the following resolution of the Annual Meeting was brought before the Parliamentary Bills Committee last week: "That in the opinion of this meeting it is expedient that an Act of Parliament should, as soon as possible, be passed providing for the registration and education of medical, surgical, and obstetric nurses; and the Council of this association are therefore requested to consider this matter, and to take such measures as may seem to them advisable to obtain such legislation." After some discussion it was resolved, "That the chairman be instructed to call a conference of the representatives of the various nursing bodies, to obtain the opinions and ascertain their views on this subject." It will be interesting to observe the attitude of the nurse training schools so approached. It is not probable that a responsible body like the Council of the British Medical Association would be content to assist the formation of any board not fully representative of those most concerned in the training of nurses Hitherto the majority of the heads of the more im portan training schools have resolutely opposed registration of any description, and even the more advanced spirits amongst them favour only the idea of some cooperative arrangement of a voluntary nature. That this would become possible in conjunction with State regulation is very doubtful. Our own convictions on the subject have been expressed frequently in these columns, and we shall watch the issue of the proposed conference with genuine interest.

### SHORT ITEMS.

Miss Jessie Cameron has been appointed nurse at the Kyle Union Poorhouse, Ayr.—The London Obstetrical Society will hold its next examination for midwives on January 6th, at eight p.m., at 27, Hanover Square.—The Tralee Board of Guardians have agreed to employ night nurses in the sick wards.—The sale of work in aid of the funds of the Birmingham and Midland Hospital for Women was a brilliant success, thanks to the energy of the ladies and gentlemen by whom the arrangements were made. The sum of £482 was raised, from which less than £60 for expenses had to be deducted.—In an interview with the lady who goes to Ashantee as head of the Army Nursing Sisters, the correspondent of the *Queen* newspaper reports her to have said, "Every Army nurse is required to have served at least a year previously in a civil hospital." This was the case some years ago, but now no nurse is admitted to the Army Nursing Service unless she holds a certificate for a complete course of hospital training.

### Elementary Physiology for Nurses.

By C. F. Marshall, M.D., F.R.C.S., late Surgical Registrar Hospital for Sick Children, Great Ormond Street.

XVIII.—THE EYE (*continued*).

*Accommodation.*—It is necessary for distinct vision that the image should fall on the retina. Now the position of the image varies with that of the object. Compare with the candle and screen; if the candle is a long way off the lens the image is formed at a definite distance from it; if the candle is moved nearer the lens the image is now further from it. For each position of the object there will be a corresponding position of the image; as the object approaches the lens the image recedes from it.

Hence, if the eye were made of glass there would be only one distance at which we could see distinctly. This is obviously not the case. We can see objects at a distance and objects close to us, but we cannot see both at the same time; there is a distinct effort in looking from one to the other, and this effort is accommodation. What happens in the eye is that the lens changes shape according to the distance of the object looked at. Suppose the lens is accommodated for a distant object, the image of a near object will be behind the retina. For a near object the eye is long-sighted, which we have seen is corrected by convex lenses. This is done in the eye itself by making the lens more convex when near objects are looked at.

The mechanism by which this is effected is thus: At the junction of the choroid and iris is the ciliary muscle, a circular ring of muscle round the edge of the iris and attached to the sclerotic. The lens is enclosed in an elastic capsule, which is always on the stretch, thus tending to flatten the lens. Contraction of the ciliary muscle relaxes the capsule of the lens, and allows it to become more spherical, and hence its anterior surface more convex (See Fig. 13, page lxxi). Thus the lens alters its degree of convexity according to the distance of the object, and can thus form objects at different distances distinctly on the retina.

There is a distinct effort necessary in order to accommodate. If we hold a book at a distance and gradually bring it nearer to the eyes, we can still see it clearly, but as it gets nearer the effort becomes greater and greater, till it becomes impossible. The near point of vision is about five inches from the eye, this is the nearest point at which objects can be seen distinctly. The far point, in a normal eye, is infinity.

*Presbyopia.*—In old age the near point recedes from the eye owing to diminution in the elasticity of the lens and loss of power of the ciliary muscle. Distant vision is, however, as good as ever. Presbyopia is corrected by using convex glasses when reading.

*Inversion of the Image.*—The image on the retina is upside down. There is no doubt about this, for we can see the image of a candle inverted by looking at a sheep's eye, with the back part of the sclerotic cut away. Why, then, do we not see objects upside down? We must not suppose that we look at the image which is on our own retina, or that the retina is the seat of vision. The brain is the seat of the sensation of vision, as of all other sensations, and stimulation of a certain part of the retina causes a message to be sent to the brain, which thus knows what part has been stimulated and no more. In fact, it is simply a matter of experience. Supposing an object lies on our right side, the image will be on the left side of the retina, and by experience we know that stimulation of the left side of the retina can only be caused by light from an object on our right side.

The infant has to learn all this for itself. Hold an object before an infant and see the unavailing efforts it makes to seize it. These are partly due to imperfect co-ordinating power over the muscles, and partly to imperfect knowledge of what its visual sensations are. Just as with sensory nerves an impression received along a particular nerve is always referred to a particular part of the body, so with the eye an impression on a certain part of the retina is referred to an object in a particular direction, because experience has shown that this must be so.

*Persistence of Images.*—The impression made on the retina does not subside at once, but lasts about one-eighth of a second on the average. In the case of very bright impressions the persistence is much longer, as we know ourselves by looking at the sun. This is the principle of the zoetrope, or wheel of life, which represents a series of figures in successive phases of the acts of running, leaping, &c. These, when made to pass rapidly before the eye, are by a mental process combined into a continuous series, owing to persistence of the images. The iris acts as a diaphragm, cutting of the marginal rays of light, and so rendering vision more distinct. The iris contain muscular fibres, and has the power of contracting and dilating. In the dark it dilates, and in the light it contracts, thus causing alteration in the size of the pupil and in the amount of light admitted to the eye.

### Where to Go.

Royal Holborn Theatre of Varieties.—A benefit has been arranged for Friday, December 13th, at seven p.m., in aid of the Western Ophthalmic Hospital. Tickets can be obtained at the theatre, or from Captain Hastings Neale, secretary, Western Ophthalmic Hospital, Marylebone Road.

Chrysanthemum Show.—The last great show of chrysanthemums this year by the National Chrysanthemum Society will be held on Tuesday, Wednesday and Thursday next, December 3rd, 4th, and 5th, at the Royal Aquarium, when an exceptional display is expected.

Trained Nurses' Club.—Massage Talks, Saturdays, at a quarter past two: December 7th, "Electricity"; December 14th, "Nauheim Treatment of Heart Disease." Admission, 1s. 6d.; members of the Society of Trained Masseuses, 1s. 3d. Cookery Talks, December 7th and December 14th, at four o'clock: "Invalid Cookery on a Spirit Stove," Miss Bessie Halliday, Diplomée School of Cooking. Admission, 1s. 6d.

Art Embroidery Show Rooms, 25, Old Bond Street.—A Christmas sale and exhibition of art embroideries is being held in these rooms. It is well worth a visit of inspection, and the Harris embroideries in flax threads on their own art linens are most beautiful. The nattiest, daintiest book covers, worked in antique designs, tea cloths, tea cosies, cushions, art linen aprons, bedspreads, counterpanes—all these are shown in many varieties of form and colour. In fact, the uses are legion to which Messrs. Harris have applied their fascinating art linens. The novelties here displayed are well suited for Christmas presents, and to limited means.

### Wants and Workers.

[The attention of correspondents is directed to the fact that "Helps in Sickness and to Health" (Scientific Press, 428, Strand) will enable them promptly to find the most suitable accommodation for difficult or special cases.]

Bundles for a jumble sale on December 20th, in aid of the funds of the Trained Nurses' Club, gratefully received by the Secretary, 12, Buckingham Street, Strand; or by the Treasurer, 28, Boltons, S.W.

### The Comforts of Nurses.

From a Working Woman's Point of View.

### A COMPARISON.

In talking of the hardship of long hours and small pay as the ordinary lot of the hospital nurse, it seems to me (who am one of the sisterhood) that there is a tendency to ignore many comforts and blessings. For example, there is the typewriter, often a woman of education and refinement, who must provide for herself during her apprenticeship, board and lodging in health, and in sickness she has an added burden of doctor's and nurse's fees, and all the costly "extras" which illness necessitates. She is a solitary atom in the working world, and if she has no home she must be her own protector and bread winner in the course of a career which she may embark upon whilst still in her teens.

### The Sick Nurse.

The hospital nurse, on the contrary, is one of a community, and has board, lodging, laundry, and uniform found for her; she has the best nursing and medical skill in the hospital placed freely at her service. Who does not know the hushed accents in which it is announced that "Poor Nurse Z has typhoid," frequently followed by the remark that "Dr. ——," perhaps one of the best known physicians in the kingdom, "came down to see her again last night; he *is* good to the nurses." The general public canonizes the nurse if she dies, and her companions are too loyal ever to hint that her own imprudence or disobedience to rules had a great deal to do with her fatal illness. But those who lament the early death of a worker, or rejoice in her perfect recovery, seldom realise that on her have been bestowed incessant care and skill such as could not be exceeded in the case of a princess. Therefore, I venture to maintain that nurses have much to be thankful for.

### Governesses and Students.

The governess, whether resident or daily, has no chance of obtaining such help, though, of course, hospital wards are open to all urgent illness. Her certificates and experience may command far higher pay than is earned by the nurse, but she has many expenses, and if she has no home her holidays consume all that she manages to save for them, her dress being a heavy item, for she is out in all weathers and must wear decent clothes. The art student, too, has to keep herself, and often finds making "two ends meet" a task beyond her powers; whilst the student of medicine has big fees to pay, expensive books to buy, and to feed, clothe, and lodge herself during five years of hard work, with very great uncertainty as regards money-making for many years afterwards.

The student, the governess, the journalist, and the typist and clerk are often sisters of nurses, and in their hearts feel how far more precarious are the callings which they follow themselves.

### Over-Care.

Were it not for a dread of being misunderstood, I should like to say sometimes that we nurses are too much looked after, and a great deal too much talked about. I begin to fear the race will degenerate under the guardianship which surrounds its every step. I own to being sadly weary of the amount of patronage, pity, and criticism with which the kindly general public favours the healthy, busy, and independent hospital nurse. Awhile back the trials of shop girls were on every tongue; barmaids and factory hands have also been discussed at great length; but for evergreen, permanent durability, the supposed woes of the trained nurse remain prominent. I grant that in some hospitals the hours spent on duty are too long, but most forms of women's work require that the best hours of life be devoted to the calling followed from taste through circumstances.

### Another Comparison.

Compare with the working hours of a nurse those of the middle-class mother and daughter, and which are the easier? The former's responsibilities cease when she leaves her ward; the latter's go on both night and day. It is only when a nurse exchanges life in a well-managed hospital for that of any ordinary household that she realises the comfort of the former in the matter of divided responsibilities.

The house mother has often as much standing about as the hospital nurse, and the elder daughter often finds life in a ward is lighter than previous days spent in working at home.

As regards the food in hospitals, it is hard to speak with decision, for no two people ever quite agree on the subject of meals. It is impossible to feed at any restaurant or tea-room without noticing that whilst half a dozen persons make no complaints, and leave cleared platters as evidence of good and wholesome appetites, similar portions are declined at the next table as "Not fit to eat" by some jaded dyspeptic or confirmed epicure. Therefore it is impossible to hope that any hospital diets will give satisfaction to every member of the staff. The cooking might doubtless be improved in most hospitals, but only plain wholesome food should be demanded by those who work in institutions.

### Annual Pay.

The rate of pay is disparaged by those who fail to understand that the first salaries are given in addition to instruction in a profitable calling, one which will never fail so long as a nurse is worthy. To be constantly told that a probationer earns servants' wages is a little bit galling to a girl, though she may proudly call herself the "Servant of the Sick." When probationership is ended the earnings increase, as is just and natural; and a salary of from £25 as staff nurse to £45 as ward sister can be looked for. I think that staff nurses should be paid £30 per annum during the fourth year of service, but I do not suppose my opinion will carry much weight, even though a personal experience of the difficulty of putting anything by out of a smaller salary tempts me to speak. Theoretically, I know, every single woman ought to make some provision for her old age, but to put this into practice necessitates such incessant self-denial as no one who has not tried it can estimate. For a hospital nurse to set aside a third of her earnings to secure herself against destitution in her old age is really an act of heroism. It means her going without pleasure, even cheap days off at the seaside or in the country, or an occasional concert or play. It means weary walks to save omnibus and tram fares, it means the abandonment of subscriptions and small private charities, and, worse still, it renders occasional present-giving impossible.

"What! give up every little pleasure till one is too old for enjoyment, just to avoid possible poverty in an old age which may never be reached!" protests the young nurse; and I find it hard to resist her plea that I, too, will "fling prudence to the winds," and join in a jaunt to the country. Of course, at some hospitals the committee are generous in personally providing free outings for nurses, but it cannot always be done, though these "extras" are much appreciated.

I do not personally know of any form of woman's work outside the nursing profession where provision is made for board, lodging, medical and nursing care, uniform, and laundry expenses for those engaged in it. When so much is freely provided, it can hardly be denied that a woman is actually better off as a hospital nurse with a moderate salary than her sister, the governess or lady clerk, who gets three times as much money, but has corresponding expenses.

### Family Claims.

I have heard objections raised to a nursing career because it cuts a woman off from her friends, but it is a little remarkable that this is seldom made a grievance of by the nurse herself. Again, some people speak of those nurses who "have to assist their parents" as if this duty were their special prerogative, instead of being one which every son and daugher in the world may, at some time or other, have to face. If I were asked to define a hospital nurse, I should say: "She is a woman who has learnt a useful profession, who has an interesting, eventful life, with limited responsibilities and unlimited opportunities.

### The Princess Maud Marriage Present Fund.

Nurses sending contributions to this Fund are requested to write outside the envelope in the left-hand corner the words "Princess Maud," as this will save considerable trouble in dealing with the correspondence. Nurses are reminded that all letters on this subject should be addressed to the Manager of The Hospital, 428, Strand, London, W.C., and not to the office of the Pension Fund.

Amount previously acknowledged ... £35 1s.

Fourth List.

| | s. | d. | | s. | d. |
|---|---|---|---|---|---|
| J. Brown ... ... ... | 4 | 0 | K. J. Chetwind ... | 1 | 0 |
| K. Nelson ... ... ... | 2 | 0 | E. Morris ... ... ... | 1 | 0 |
| F. Robinson ... ... | 2 | 0 | E. Butler ... ... ... | 1 | 0 |
| Nurse West... ... ... | 1 | 0 | M. E. Dunn ... ... | 5 | 0 |
| Policy No. 3,114 ... | 1 | 0 | A. R. Webster ... ... | 2 | 6 |
| E. A. Norton ... ... | 1 | 0 | M. Manwaring ... ... | 1 | 0 |
| E. Wilson ... ... ... | 1 | 0 | H. Flint ... ... ... | 1 | 0 |
| H. Smith ... ... ... | 1 | 0 | Policy, No. 2,144 ... | 2 | 6 |
| Policy No. 405 ... ... | 1 | 0 | C. M. Martin ... ... | 1 | 0 |
| A. Bond ... ... ... | 1 | 0 | Policy, No. 4,231 ... | 1 | 0 |
| E. Boul ... ... ... | 1 | 0 | B. Collins ... ... ... | 1 | 0 |
| Nurse Packwood ... | 1 | 0 | M. Wakeford ... ... | 1 | 0 |
| S. Boorman ... ... | 1 | 0 | Policy, No. 1,399 ... | 2 | 0 |
| E. Myers ... ... ... | 1 | 0 | Nurse Seabrook... ... | 1 | 0 |
| E. Ussher ... ... ... | 1 | 0 | M. Storey ... ... ... | 1 | 0 |
| E. C. H. Jackson ... | 1 | 0 | E. S. Barnes ... ... | 1 | 0 |
| C. F. Baillie ... ... | 1 | 0 | Policy, No. 201... ... | 1 | 0 |
| S. A. Buckley ... ... | 1 | 0 | M. C. Bezant ... ... | 1 | 0 |
| E. Trott ... ... ... | 1 | 0 | J. L. Wilson ... ... | 1 | 0 |
| Nurse Balderson ... | 1 | 0 | M. B. Eatall ... ... | 1 | 0 |
| M. Frewson ... ... | 2 | 0 | A. Badcock... ... ... | 1 | 0 |
| A. J. Little ... ... | 2 | 0 | A. Spring ... ... ... | 1 | 0 |
| E. J. Aston... ... ... | 1 | 0 | Nurse Cockcroft ... | 1 | 6 |
| F. A. Edwards ... ... | 1 | 0 | F. Ordish ... ... ... | 1 | 0 |
| F. C. Dell ... ... ... | 1 | 0 | E. E. Reynolds... ... | 2 | 0 |
| Policy No. 1,459 ... | 1 | 0 | M. Percival ... ... | 1 | 0 |
| E. Proviss ... ... ... | 1 | 0 | A. Purchall ... ... | 1 | 0 |
| M. A. Tomkins ... ... | 1 | 0 | Anon. ... ... ... ... | 1 | 0 |
| M. E. Jones ... ... | 1 | 0 | B. A. Heath ... ... | 1 | 0 |
| A. L. Church ... ... | 1 | 0 | M. Nicholson ... ... | 1 | 0 |
| E. B. G. Gray ... ... | 1 | 0 | F. L. Forbes ... ... | 1 | 0 |
| A. M. Fox ... ... ... | 2 | 0 | S. W. Doughty ... ... | 2 | 0 |
| E. E. Neate ... ... | 1 | 0 | M. E. ... ... ... ... | 2 | 0 |
| A. Blackwell ... ... | 1 | 0 | E. Gold ... ... ... | 1 | 0 |
| Nurse Cripwell ... ... | 1 | 0 | A. N. Lander ... ... | 5 | 0 |
| E. Sanford ... ... ... | 1 | 0 | E. Tobias ... ... ... | 1 | 0 |
| Policy No. 1,320 ... | 2 | 6 | A. Haig-Brown ... ... | 1 | 0 |
| M. Thomas... ... ... | 1 | 0 | F. Godrich ... ... ... | 2 | 0 |
| G. Digwood ... ... | 1 | 0 | N. Frost ... ... ... | 1 | 0 |
| E. Smith ... ... ... | 1 | 0 | Policy No. 2,642 ... | 1 | 0 |
| G. Mannox... ... ... | 1 | 0 | M. Scott ... ... ... | 1 | 0 |
| A. J. Kingsbury ... | 1 | 0 | F. Macdonald ... ... | 1 | 0 |
| A. L. Read ... ... ... | 1 | 0 | A. Deville ... ... ... | 1 | 0 |
| T. B. Anderson ... ... | 2 | 6 | V. F. Simpson ... ... | 1 | 0 |
| Nurse Kimber ... ... | 1 | 0 | E. Graham ... ... ... | 2 | 0 |
| A. M. Lane... ... ... | 5 | 0 | Nurse Coleman ... ... | 1 | 0 |
| J. Walker ... ... ... | 2 | 0 | Policy No. 100 ... ... | 1 | 0 |
| A. Cottle ... ... ... | 1 | 0 | E. A. Dowse ... ... | 1 | 0 |
| G. Jones ... ... ... | 1 | 0 | J. Hoadley... ... ... | 1 | 0 |
| Miss Corneille ... ... | 1 | 0 | M. E. Harper ... ... | 1 | 0 |
| A. W. Messum ... ... | 2 | 6 | E. Parkinson ... ... | 1 | 0 |
| D. L. West... ... ... | 1 | 0 | S. A. Pitchford... ... | 1 | 0 |
| M. Foster ... ... ... | 1 | 0 | E. J. Messenger ... | 1 | 0 |
| A. M. Browne ... ... | 1 | 0 | Nurse Stirling ... ... | 2 | 0 |
| L. Webb ... ... ... | 1 | 0 | C. Kennedy ... ... | 2 | 0 |
| Policy. No. 1,296 ... | 1 | 0 | E. W. ... ... ... ... | 1 | 0 |
| A. Headford ... ... | 1 | 0 | A. R. ... ... ... ... | 1 | 0 |
| A. M. Warren ... ... | 1 | 0 | E. A. ... ... ... ... | 1 | 0 |
| Policy, No. 1,490 ... | 2 | 6 | Nurse Hislop ... ... | 1 | 0 |
| A. Robinson ... ... | 1 | 0 | L. M. Lee ... ... ... | 1 | 0 |
| G. Kinninmont... ... | 1 | 0 | S. Bardwell ... ... | 1 | 0 |
| Policy, No. 3,004 ... | 1 | 0 | | | |

Received per Royal National Pension Fund.

| | s. | d. | | s. | d. |
|---|---|---|---|---|---|
| Nurse Inkson ... ... | 1 | 0 | M. Elsby ... ... ... | 1 | 0 |
| L. Roach ... ... ... | 1 | 0 | Miss Collinson ... ... | 1 | 0 |
| B. E. Shaw ... ... ... | 1 | 0 | L. Stutter ... ... ... | 1 | 0 |
| Miss Cawte... ... ... | 1 | 0 | F. M. Hewitt ... ... | 1 | 0 |
| L. Hill... ... ... ... | 1 | 0 | S. Barnes ... ... ... | 1 | 0 |
| L. Gunn ... ... ... | 1 | 0 | F. C. Pocock ... ... | 1 | 0 |
| B. Aitken ... ... ... | 1 | 0 | A. Purcell ... ... ... | 1 | 0 |
| E. Manley ... ... ... | 1 | 0 | | | |

### Nursing in Florence.

### II.—SPEDALE DE BONIFAZIO.

This immense hospital is a succursal of Santa Maria Nuova, and under the same administration, though infinitely superior in every particular. It was founded in the fourteenth century, and consists of an old and a new part. It derives a large income from the legacy of a man called Galli, who left a sum of money to several cities of Italy for hospitals, and the share Floren ce was entitled to came to 700,000 francs (or £28,000).

Even in the old part, which is by no means perfect, each bed has a basket at the head, where the patient may keep any little private possessions, so that they need not adopt the sickening practice of putting food under their pillows or their backs when brought by their friends. The patients wear stone-coloured nightgowns, which are not at all becoming, and the wards are inadequately heated with burning charcoal in huge pans. The ophthalmic wards struck us as especially cold.

The new part of the hospital is a great improvement on the old. It is built on the pavilion system, connected by pretty covered corridors airy and bright; the wards are large, but not unpleasantly so, and though lacking the home look of ours, have the advantage of the Italian sun, which is allowed to pour in unchecked unless it inconveniences the patients. There are excellent ventilators, so that even when the windows are shut there is no lack of fresh air. The mattresses are of wool, and good of their kind. At the time of our visit the wards were lighted by petroleum lamps, but the authorities hoped shortly to have electric light or gas. All the wards in the new part were clean and without smell, and the closets well supplied with water. All were on the same principle, viz., with an isolated room at one end, and a good lavatory and bath at the other.

Every six hours there is a change of Franciscan sisters in charge, but here, as in the mother hospital, there are far too few of them. The doctors visit the patients twice a day, and one is always on the spot in case of emergency. In the convalescent ward each patient has his or her own towel and drinking glass and a little table at the bottom of the bed.

Soiled linen is passed down shafts; so also are the sweepings of the wards. The washhouse is worked entirely by paid labour. The kitchen is large, clean, and businesslike. Eighty kilos of meat are used daily for beef-tea, which is very good indeed; and the meat for the patients is sweet and tender. The dispensary is complete in all respects, and is for this hospital alone; and the clergy of all denominations are allowed to visit.

Since 1866 no insane have been received. Incurables brought in from S.M. Nuova are taken gratuitously, but if from their own homes one franc thirty-five centimes is charged daily. Dr. Nesti was our guide through this hospital. The bath establishment belonging to it is the most perfect we have ever seen, and deserves a separate description.

### Royal British Nurses' Association.

The following additional railways will generously grant return tickets for single fares to members attending the conversazione on Monday, December 9th, such tickets being available from December 7th to 10th: Great Western, London and South-Western, London, Chatham, and Dover, and London, Brighton, and South Coast. This concession will be made only on presentation of the tickets of admission to the conversazione at the respective booking offices, and, if possible, the card of membership also.

### Everybody's Opinion.

[Correspondence on all subjects is invited, but we cannot in any way be responsible for the opinions expressed by our correspondents. No communications can be entertained if the name and address of the correspondent is not given, or unless one side of the paper only be written on.]

### REAL AND SHAM NURSES.

"POLICYHOLDER 1,399" writes: In THE HOSPITAL Nursing Supplement a correspondent asks for suggestions as to a remedy for the present abuse of nurses' uniform. I do not see that anything can be done to prevent a woman wearing whatever dress she chooses, but surely nurses have one remedy in their own hands. Let every nurse join the Pension Fund. This may be considered a guarantee that the policyholders are genuine nurses. And all have the privilege of wearing the Princess's armlet when they become members of the Fund.

### THE NURSES' DRESS.

"A LOVER OF THE BONNET AND CLOAK" writes: As a wearer for nearly sixteen years of nursing uniform, may I be allowed to say that I think the suggestion made by "A Provincial Trained Nurse" a most sensible one? An appeal to "Our Princess" could but result in good of some kind. I wonder if my sister nurses think it feasible to petition "Our Princess" to design or cause to be designed a bonnet and cloak to be worn by all her own nurses? Could not such a design be made copyright or patented or something of that sort, and so render it illegal for any but Our Princess's own nurses to wear it? I am an ignoramus in legal matters, but "our best friend" could perhaps tell us whether such an idea is likely to succeed? That such a uniform would be worn with pride and satisfaction by all Pension Fund nurses goes without saying, and I am sure we can trust "Our Princess" to approve only a design that is dainty and becoming to loyal and grateful nurses. The uniform bonnet and cloak save both time and money, to say nothing of their comfort.

### WORK IN ASYLUMS.

"A LATE ASYLUM AND PRESENT HOSPITAL NURSE" writes: At a time when there is so much discussion on asylums and their management, I should like to give my experience as a nurse who was for upwards of three years in a large county asylum in the North of England. During that time I found medical officers, head attendants, and deputies very considerate to the staff. Our duties commenced at six a.m., and lasted till quarter-past seven and half-past eight p.m., with one ten o'clock night in a week. Breakfast was served at seven a.m., and consisted of good bread and butter, not brown bread and margarine, but the tea was certainly often lukewarm and of inferior quality. Dinner was too much in amount, but badly cooked and served up. Five o'clock tea was precisely the same as breakfast. In the infirmary the nurses dined in a room adjoining the wards, not in close proximity to the patients. The patients had to be taken out in hot and cold weather, but there were sufficient nurses for the patients, and others near at hand in case anyone became rough and violent, which was by no means a daily occurrence. On occasions when anything did happen the nurse always had the sympathy of her fellow nurses, and of medical officers and head attendants. Everyone knows that nurses and attendants have lost their lives when discharging their duties, and it is very sad to think about, but from my own experience personal injury is certainly not an everyday occurrence. Our sleeping arrangements were not so good as they might have been, but many successive sleepless nights were unknown. When new admissions were excited and noisy, or old ones either, then nurses sleeping near the refractory wards were disturbed, but it was not a constant thing, and I cannot believe that anyone is so inhuman as former correspondents would have us believe. There was a Protestant and a Roman Catholic chapel, and all followed their chosen worship. Sometimes a Protestant was asked to take a Roman Catholic to church, and *vice versa*, but this was the exception, not the rule, as it was an understood thing for the charge nurse to arrange for those under her care to go to their own church and with patients of their own denomination. I must say that I think previous letters on this subject are calculated to give a very wrong idea of asylum life. Not that I wish to convey the impression that it is all *couleur de rose*, or that I would sacrifice my own training to take any asylum post which was offered me. I think any girl who has herself to support might do much worse than go into one of those institutions. If she is the educated and refined type of woman wanted as nurses, she need have no misgivings about the respect that will be accorded her from those in authority. Can these nurses who complain offer any practical suggestions? For myself, I should say let our asylums be worked more on hospital lines. The plan that is steadily increasing of filling the more responsible posts with trained nurses is to be highly commended. I should like to hear such a one's views on the subject, especially if she has taken up asylum work recently.

### NURSES IN IRISH ASYLUMS.

"W. E. E." WRITES: In reply to the Editor's question as to why I considered that the nurses in Irish asylums fared better than those in England, I may state the nurses did not sleep in wards with the patients; there each senior nurse had a room to herself, whilst the others had comfortable dormitories safe away from the patients. They also had a nice little sitting-room, provided with a piano, and a comfortable mess-room. Special attention was paid to the nurses' dietary, a cook being told off especially for them, and their meals were well cooked and well served. The medical superintendent left no stone unturned to provide for the comfort of his nursing staff, and, in return, he got good servants, whose services were appreciated. This gentleman was English, and it would be well if a few more English doctors were like him.

### CHRISTMAS DECORATIONS.

THE HON. SYDNEY HOLLAND (Chairman of the Poplar Hospital for Accidents) writes: You have done good service in drawing attention to the growing custom of decorating hospital wards at Christmas time. I write "growing," because every year the decorations seem to me to be more elaborate. There is not the slighest doubt that making these decorations, putting them up, and taking them down mean a great deal of extra time in the wards to the sisters and nurses who should be off duty and out of the wards. This in itself is objectionable, apart from the real manual work involved in making them, and apart from the point to which you refer that the decorations often cost the medical and nursing staff considerable sums. There cannot be the slightest doubt that decorations are objectionable from a sanitary point of view. Once up they are invariably left up too long. None likes to run the risk of being thought a "wet blanket," especially at Christmas time, and so those in authority hesitate to say "No decorations." But at the Poplar Hospital the suggestion was made (with fear and trembling) that no ward should be decorated, and it has been most cordially welcomed by both medical and nursing staff. Some people are sure to say, "How dull!" but they will be people who stroll into the wards at Christmas time (not on Christmas Day) to bless us all with their smiles and approval, and never come at any other time. Too much attention need not be paid to their remarks after they have gone. Christmas can surely be made "happy" without our walls being covered by day and night with decaying vegetables.

### The Medical Study for Women in Russia.

Up till the year 1882 there had, for some time, been what were called "female doctor classes" at the Nicolay Military Hospital, which, however, were terminated by an Imperial decree of August 5th in that year. Now the women of Russia have again obtained the right to study medicine in their own country, a Female Medical Institute being in course of completion in St. Petersburg. The authorities have persistently refused to grant women permission to study at the Russian universities together with male students, and the new institution only gives ladies access to the study of medicine. The instruction will more especially comprise female and children's diseases and accouchement. Only women of the Christian faith will be received, and Jewesses are consequently not admissible; the age is limited between twenty and thirty-five years. The number of lady students can be limited according to order from the Ministry for Instruction. The applicants must be able to produce certificates from the police of being politically harmless, and they also require a written permission from parents, guardians, or husbands. The course at the Institute extends over four years, divided into eight terms from August 20th to December 20th and January 15th to May 30th respectively. After this four years' course the students will have to practically perfect themselves during a period of from one to three years, by attending lying-in establishments and special hospitals for women and children, under the guidance of experienced doctors. The students who have satisfactorily passed the requisite tests and examinations obtain a diploma as "female doctors." This diploma gives them the right to practise everywhere in Russia, and to accept posts as doctors at female charitable and training institutions, at hospitals for women and children, and under the hygienic police, but not in posts for which the appointments are made by the State. In the rural districts the local medical authorities can give them certain posts at hospitals and as "district doctors" or officers of health.

A home will be connected with the Institute where such students can live as have no parents or near relatives in the town. The annual fee for the instruction is 100 roubles. The building is to be ready and the Institute to be opened in the course of the summer 1897. The necessary funds have been collected by voluntary donations, and the city of St. Petersburg will give an annual grant. It is impossible to foretell the probable number of lady students, but it is likely to be very considerable indeed.

### Appointments.

Stratford-upon-Avon Joint Infectious Hospital.—Miss Christine Aldis has been appointed Matron of this hospital.

Coatbridge Fever Hospital.—Miss M. A. Hempseed has been appointed Matron of this hospital. She was trained at Greenock Infirmary and Glasgow Maternity Hospital, and joined the staff of the Glasgow Sick Poor and Private Nursing Association. In 1893 Miss Hempseed was appointed a Queen's Nurse, and became entitled to wear the badge and brassard granted by the Queen Victoria Jubilee Institute. We wish Miss Hempseed every success in her new post, and congratulate her on her testimonials.

Chertsey Union Infirmary, Ottershaw, Surrey.—Miss K. E. Richmond, who was trained by the Workhouse Nursing Association, has been appointed Superintendent of Nurses at Chertsey Union Infirmary. Miss Richmond has had valuable and varied experience, having worked for two years in Edinburgh Royal Infirmary, one year at Hartlepool Union, about sixteen months at Paddington Infirmary, at Leamington Home for Incurables from May, 1893, to July, 1895, and at Kensington Infirmary from then to October last. We wish Miss Richmond every success in her appointment. She will be assisted by six nurses, also from the Workhouse Nursing Association.

### Asylum Lectures.

DUNDEE ROYAL LUNATIC ASYLUM.

Lectures to nurses and attendants are given once a week during the winter months by Dr. James Rorie.

Lecture I., "On General and Special Duties"; II., "Duties in Special Cases"; III., "On the Human Body"; IV., "The Cavities of the Body and their Contents"; V., "On the Brain, Nervous System, and Special Senses"; VI. and VII., "The Mind or Intellectual Principle"; VIII., "Man's Moral Nature"; IX., "The Principle of Justice"; X., "The Forms of Mental Diseases met with in Asylums."

Practical lessons are also given in Bandaging, Ordinary Nurse Work, Preparation of Linseed and Mustard Poultices, Application of Fomentations, &c.

Dr. Rorie has lectured for the past ten years to his staff, and last winter he arranged for a course of instruction in sick cookery, which proved a source of great interest to the pupils, and a valuable part of asylum nurse training.

### Notes and Queries.

### Queries.

(44) *Training.*—What premium is required for training at Queen Charlotte's Hospital, and how much uniform do pupils have to provide?—*Nora.*

(45) *Mesmerism.*—*Inquirer.*

(46) *Employment.*—I want to be advised as to getting employment. I was trained at the British Lying-in Hospital three years ago.—*Nurse Dorothy.*

(47) *Nurse.*—Can a nurse keep a mental case in her house without being certified?—*E. R.*

(48) *Midwifery.*—Is there a qualification to practise midwifery only?—*Medicus.*

(49) *District.*—Can you tell me where to get district training? I have applied to several institutions, and have been offered thirteen weeks' training for thirteen guineas, but want to know if a certificate gained by such means could ever justify me in calling myself a district nurse? I have so far only had maternity training, and find it difficult to get on without general experience.—*S. E.*

(50) *Home.*—Can you tell me of a home where a girl of twelve, lame through rheumatism, could be trained to earn her own living? She has another troublesome ailment.—*M. M.*

(51) *Pattern.*—Where can the exact pattern of the aprons worn by the sisters and staff nurses at the London Hospital be obtained?—*Nurse B.*

(52) *Matrons.*—Will you kindly tell me the proper length for a matron's holiday?—*S. B.*

### Answers.

(44) *Training (Nora).*—Write and ask the matron of the hospital. It is in Marylebone Road, London.

(45) *Mesmerism (Inquirer).*—Your question is not one which could be answered in this column.

(46) *Employment (Nurse Dorothy).*—In answer to your inquiry a correspondent has courteously made the following suggestion, "Would you care about the duties of a cottage nurse? They are to nurse the sick poor in their homes; to live in their cottages while nursing them. In confinements to perform such duties as are required in the cottage, cooking the dinners and sending the children to school, &c., besides nursing the mother and infant. Eight shillings a week for the first year and uniform, and ten shillings a week for the second year and uniform."

(47) *Nurse (E. R.).*—She can take in anyone as a lodger, but she cannot exercise any control over such a person.

(48) *Midwifery (Medicus).*—There is no recognised qualification to practise midwifery only. The General Medical Council will not now permit anyone to be registered who is not qualified in all three subjects—medicine, surgery, and midwifery. If our correspondent is a young man we cannot advise him to enter on the life of an unqualified assistant. The action of the General Medical Council, in regard to what is termed "covering," tends very much to lessen the demand for assistants who are unqualified.

(49) *District (S. E.).*—We fear from your letter that your age now prevents your going in for hospital training. Have you thought of applying at a good fever hospital, where you might be eligible for a two years' course? There is good work to be done there, and in nursing small-pox also. If you prefer district work you must content yourself to become only a "cottage" or "village" nurse, and certainly must not adopt the title of the thoroughly-trained district nurse. It may help you to read the following extract from Miss Le Pelley, of Ho[illegible]ham, who has written respecting another correspondent of ours whose previous training has been somewhat similar to your own: "The Committee are agreeable to take her and give her training in general work. She will then only give six weeks for her training." [At the end of that time payment at the rate named in answer given to Nurse Dorothy in this column is promised.] "The work is hard, but the nurses find it very interesting, and they are much liked. The work of cottage nurses is to nurse the sick-poor, though very often they attend the middle-class." After this, six weeks' training in general district work. We are assured by Miss Le Pelley that "cottage nurses are not sent to serious operation cases beyond their skill."

(50) *Home (M. M.)*—It seems to us that the girl ought to undergo proper medical treatment in the first place.

(51) *Pattern (Nurse B.)*—If you write to the matron you will be sure to get a courteous reply.

(52) *Matrons (S. B.)*—Every matron should have at least a month's holiday annually. When the position is a very responsible one it is usual for a committee also to grant a few days' extra leave now and then.

### Dundee Royal Lunatic Asylum.

RECIPES FOR INVALID COOKERY.

I.—Toast Water.—One pint of boiling water is allowed to cool, and to this is added 2 oz. bread slowly toasted till buite crisp and brown, but not blackened or burned. Allow to stand one hour, and strain through muslin. Should never be used after standing 12 hours.

II.—Blanche Eau.—Put a handful of oatmeal into a jug or bowl. Pour on a pint of boiling water, and add a pinch of salt, and milk to taste. As soon as the oatmeal subsides it is fit for use.

III.—Linseed Tea.—Put half an ounce whole linseed into a covered jug. Add a pint of boiling water, and allow to infuse for two hours.

IV.—Oatmeal Gruel.—Put three tablespoonsful of oatmeal into a bowl. Mix into a thin paste with cold water. Add gradually a pint of boiling water, stirring all the time. Allow oatmeal to subside. Strain through muslin, and boil for 25 minutes. Milk may be added according to taste.

V.—Barley Water.—Put an ounce of pearl barley into a pan with half a pint cold water, and bring rapidly to boiling point. Pour off dirty water. Add two pints boiling water, and boil for two hours and a half, or until quantity is reduced to one-half. If patient may take acids, add strained juice of lemon. Lemon not to be given if patient is nursing.

VI.—Pure Milk.—Milk used for invalids should always be fresh and pure; and if it has to be kept in warm weather should be at once brought to boiling point (scalded), and allowed to cool.

VII.—Milk and Lime Water.—This mixture should for ordinary cases be in the proportion of two of milk to one of lime water. Lime water is made by slaking a piece of quicklime, and adding cold water, and keeping in closed jar. When required, remove scum from surface and decant clear liquid.

VIII.—Milk and Lime.—When a stronger alkali is required add as much chalk in powder as will lie upon a sixpence to every half pint of milk.

IX.—Beef Tea.—Cut up a pound of lean beef into small pieces the size of a bean. Put these into a covered jar, with two of pints cold water and a pinch or two of salt. If not immediately required, let it stand for an hour; then warm up gradually, and allow it to simmer for two hours, care being taken never to allow it to boil. Some prefer to add the salt after it is prepared. A large earthenware jar in a pot of warm water, like the arrangement of a glue pot, answers admirably.

X.—Strong Soup for Invalids.—Half a fowl, a knuckle of veal, a pound of neck of mutton, lean and all very fresh, are cut up into small pieces. Put into a pan with three pints of cold water, with a saltspoonful of salt. Heat up gradually, and allow to simmer gently for from four to six hours, skimming often. Then strain, and allow to stand all night, to remove fat. Twenty minutes before serving, moisten a teaspoonful of Du Barry's Revalenta Arabica, corn flour, or arrowroot, with a wineglassful of cold water, and stir into half pint of the soup. Boil slowly for 20 minutes.

XI.—Mutton Broth.—Cut up one pound lean mutton into small pieces, size of a hazel nut. Put these into two pints of cold water, and allow to simmer gently for three hours. Take off the scum as required, and add a pinch of salt. Strain off fluid, let it stand till cold, and remove fat. A dessertspoonful of Patna rice and some finely minced parsley may in some cases be added.

XII.—Veal Soup.—Prepared in same manner as mutton broth, but with a knuckle of veal and a table-spoonful of whole rice. When ready, strain and pour into a dish in which an egg has been previously beat up.

XIII.—Chicken Soup.—Choose a young bird, and, after disjointing it, boil to shreds in two pints water for about two hours. Let it cool, and skim before serving. A tablespoonful of rice may be added, and in some cases parsley.

XIV.—Oatmeal Porridge.—Add a little salt to water, and when boiling add the oatmeal by degrees, stirring all the time, then boil for 20 minutes or half an hour.

*(To be continued.)*

### For Reading to the Sick.

PATIENCE.

**Motto.**

He is not truly patient who is willing to suffer only so much as he thinks good, and from whence he pleases.—*Thos. à Kempis.*

**Verses.**

He doth not bid thee wait,
Like drift wood on the wave,
For fickle chance or fixed fate
To ruin or to save.
Thine eyes shall surely see
(No distant hope or dim)
The Lord thy God arise for thee;
"Wait patiently for Him."
—*Frances Ridley Havergal.*

Safe to the hidden house of Thine abiding
Carry the weak knees and the heart that faints,
Shield from the scorn and cover from the chiding,
Give the world joy, but patience to the saints.
—*F. Myers.*

Bide thou thy time!
Watch with meek eyes the race of pride and crime,
Sit in the gate, and be the heathen's jest,
Smiling and self-possest.
Oh, thou to whom is pledged a victor's sway,
Bide thou the victor's day.
—*Newman.*

Hast thou gone sadly through a dreary night,
And found no light,
No guide, no star, to cheer thee through the plain,
No friend, save pain?
Wait, and thy soul shall see, when most forlorn,
Rise a new morn.
*Adelaide Proctor.*

**Reading.**

Those things that a man cannot amend in himself or in others he ought to suffer patiently, until God order things otherwise.—*Thomas à Kempis.*

Observe how much easier it is to see and feel God's hand in some kinds of sorrow, especially the graver troubles of life, than it is in others. . . . The perpetual every-day demands upon our patience, our long-suffering, our forgiveness, our self-sacrifice, by response to which alone can our homes be peaceful and love-illumined. Are they not, one and all, drops of the sacramental cup of affliction, appointed by our Father's love for our strengthening and purification? It is just their humiliating littleness that adapts them most perfectly to His holy purposes. . . . The great sorrows have their special work to do; but the lesser pains and trials, the weariness of waiting when the pain itself is stilled, the wounds of unkindness . . . these finish the work of God and add peculiar graces of refined expression to the soul's character."—*E. B. Ottley.*

If when ye do well and suffer for it ye take it patiently, this is acceptable before God.—1 *Peter* xi. 20.

### How the Public can Protect Itself.

A woman travelling in an omnibus the other day was overheard to say that she was engaged in nursing a bad case of small-pox. A correspondent writing to the daily Press concerning the matter says that two of the passengers immediately left the vehicle. Unfortunately no one appears to have realised that the nurse's name and address should have been at once demanded and forwarded, with the conductor's number, to the sanitary authority. A summons could then have been taken out under the provisions of the Public Health Act, and an adequate penalty inflicted by the magistrate, with the view of protecting passengers in public conveyances.

### The Book World for Women and Nurses.

[We invite Correspondence, Criticism, Enquiries, and Notes on Books likely to interest Women and Nurses. Address, Editor, The Hospital (Nurses' Book World), 428, Strand, W.C.]

How to Become a Nurse and How to Succeed. By Honnor Morten. (Scientific Press, 428, Strand, London.)

The publishers of this already well-known guide to nursing are to be congratulated on the appearance of the third edition, for both paper and cover are decided improvements on former issues. The book is already too popular to need any special introduction, and the biographical sketche. of some of the pioneer nurses are interesting alike to the new probationer and the certificated nurse. The book is a practical adaptation of all kinds of knowledge to the needs of the would-be probationer, and doubtless in the next edition care will be taken to correct the names of the matrons of various training schools up to date. It would make the lists much more valuable if the reigning matrons' names replaced those of ladies either dead or working in other fields.

Primer of Hygiene. By Ernest S. Reynolds, M.D.Lond. M.R.C.S., Senior Physician to the Ancoats Hospital, Manchester. With fifty illustrations. (London: Macmillan and Co.)

Messrs. Macmillan, who have fully realised that a man who does not know a subject thoroughly cannot deal with it simply, and that a primer may be more difficult to write than a quarto, have, in this "Primer of Hygiene," added another to their admirable series of little books. Dr. Reynolds' volume contains no new facts and advances no new theories, but it states in clear and precise language all concerning health in the house, its situation and building, in food and in personal habits, that it concerns the average man to know. The chapter on infectious disease and disinfection and the appendix on medical and surgical emergencies are clear and practical, as we should expect from the author's position, and altogether a great deal of valuable information is conveyed in the space of 150 small pages.

One Never Knows. By F. C. Philips. (London: Ward and Downey. 1893.)

"One Never Knows" is a most appropriate title, inasmuch as one never would know why such a talented authoress as F. C. Phillips should extend such an amount of rubbish over three volumes. General inconsistency is rife throughout its pages. For instance, the heroine, Joyce Merrion, though she dances in tights in the burlesque at the Hilarity Theatre, and consents to marry secretly a roué of the aristocracy—Lord Sidney Le Brun—nevertheless is depicted as a type of all that is pure and beautiful in woman. Then, again, it hardly seems probable that the heartless, cynical man of the world should, without a thought of the future, sacrifice his fiancée—an heiress of considerable position—and jeopardise his prospects for a passing fancy. The authoress descends to a more human level when she describes the wretched life this absurd couple pass in Cape Town, where the august father has ordered them to live. It is only natural that the man should take to gambling, and ruin ensue, which Joyce, as becomes a heroine, gallantly strives to avert by again going on the stage. But at this point his old fiancée, Lady Rose, appears—now conveniently a widow—and Captain Forrester, the faithful male friend; varied complications arise; traps are set by the wicked husband and revengeful fiancée to ruin the reputation of the stainless Joyce. Yet there is no scene worthy of description. It is melodrama certainly, but of a most mediocre type. The heroine is too unnatural to be interesting, whilst the remaining parties are too vulgar to figure even in a shilling shocker.

"The Gentlewoman" Handbook of Education: What a Parent Should Know. By "Domine" (Miss Mabel Hawtrey). Edited by J. S. Wood, Editor of "The Gentlewoman." Price, one shilling.

"There's nothing new under the sun"; but there are comparatively few people nowadays who make use of the good old truths. The reason why we have to be most thankful to "Domine" is that she has collected many of these well-tried rules, and presented them to us in a charmingly fresh dress for the use of parents and guardians. Among the former—and it is chiefly for these she professes to be writing—we meet many who have most excellent ideas with regard to education and moral training of their little folk; but, though there is honest and eager desire to carry these into practice, it becomes impossible to do so, for as soon as a working plan is sought, the airy fancies on which in many cases equally airy castles have been built seem to melt away, and the children who were to have surpassed all others end by being allowed to drift into the first course which presents itself. As we read "Domine's" book, we recognise our own ideas, or ones very similar, no longer flitting and uncertain, but gathered together—classed, emphasised, and written down clearly for all who wish to profit by her kindly advice on subjects of which she is an eminently qualified judge; and imperceptibly drawn from one page to another, we acknowledge the undeniable truth of all she says, and sometimes we call to mind cases where neglect of the "small things," on the importance of which she lays particular stress, has led to the very result against which she warns us.

The last sixty or seventy pages are taken up with hints as to the choice of schools for both boys and girls, and this chapter is completed by a list of colleges and schools, each name being accompanied by a short explanatory note of the chief characteristics of the establishment, the name of its head master, the terms, &c., and other useful facts.

In cases of boys destined for the army or navy she gives all the information necessary, and in a simple and straightforward way which leaves no room for misunderstanding.

Most books of the kind are compiled primarily as guides to head masters and other members of the scholastic world, and the cut and dry statistics seem forbidding to outsiders who are less accustomed to reduce all subjects to nutshell knowledge; her book, therefore, appeals in the first instance to the pioneers of that home influence which should become the mainspring of so many individual lives, but at the same time it is none the less useful to all who are engaged in training the young.

The Romance of an Empress—Catherine II. of Russia. From the French of R. Waliszewski. (2 vols. Heinemann.)

It is only of late, M. Waliszewski informs us, that the materials necessary for the compilation of a comprehensive work on the life and character of Catherine II. of Russia have been attainable; and, even as it is, there are many State papers which are not forthcoming to the compiler of books. Doubtless, had he been able to obtain additional material, M. Waliszewski would have made good use of it. His study of a woman of whose work and character—beyond a vague idea that she was powerful and unscrupulous—most Englishmen have but little knowledge is interesting throughout. Married at fifteen to the half-idiotic Peter, living in the midst of semi-barbarism and vice, Catherine gradually developed those qualities—good and evil—which made her one of the most prominent sovereigns in Europe. Unscrupulous and wicked she was; but after reading M. Waliszewski's book one is half inclined to be astonished that she was not even worse, the nature of her life and surroundings during her youth being duly considered. That page of history which deals with her reign is, in many respects, a black one; and the light turned upon it in "The Romance of an Empress," interesting as it is, does not tend to show it in any fairer colours.

